# Programme providers’ perceptions of local variation in childhood vaccination uptake in a socioeconomically disadvantaged UK region: a qualitative study

**DOI:** 10.1136/bmjph-2026-005188

**Published:** 2026-10-22

**Authors:** Amber Sacre, Adam Todd, Clare Bambra, Sarah Sowden

**Affiliations:** 1 Population Health Sciences Institute, Newcastle University, Newcastle upon Tyne, UK; 2 National Institute of Health and Care Research (NIHR) Applied Research Collaboration (ARC) North East and North Cumbria (NENC), Newcastle upon Tyne, UK; 3 School of Pharmacy, Newcastle University, Newcastle upon Tyne, UK; 4 Newcastle Patient Safety Research Collaboration (PSRC), Newcastle University, Newcastle upon Tyne, England, UK

**Keywords:** Public Health Practice, Qualitative Research, Sociodemographic Factors, Vaccination

## Abstract

**Introduction:**

Within a global context of declining vaccination uptake, particularly in more socioeconomically disadvantaged communities, this study examined for the first time what, if anything, can be learnt from the North East of England, which, despite being the most socioeconomically disadvantaged English region, has consistently achieved high coverage of childhood vaccinations.

**Methods:**

This study aimed to explore programme providers’ perceptions of how the organisation, delivery, commissioning, support and monitoring of childhood vaccination may contribute to comparatively high uptake in the North East of England. Between June 2023 and December 2023, we conducted online semi-structured interviews with 15 professionals from across the health and care system. The transcripts were analysed using a framework approach.

**Results:**

Overall, interviewees felt that the high childhood vaccination coverage in the North East of England was explained not by the approach to provision but by the region’s sociocultural, demographic and healthcare system characteristics. The North East region was hypothesised to be more stable and less mobile in terms of both its population and healthcare workforce; conditions under which trusting parent–vaccinator relationships are more easily built and maintained. Despite this, general practice staff working in areas of high socioeconomic disadvantage described challenges, including families with complex personal circumstances and parents not viewing vaccination as a priority. In these areas, it was felt more resources were required to achieve and maintain uptake levels. Interviewees offered broader reflections on the growing complacency regarding the potential health impact of vaccine-preventable diseases and the importance of trusted voices in addressing parents’ vaccination concerns.

**Conclusion:**

Action must be taken to improve healthcare workforce stability, as a prerequisite to repairing parent–vaccinator trust. Trust is important for ensuring that parents view vaccination as a priority and for addressing vaccine hesitancy. Vaccination uptake patterns appear to be substantially influenced by social processes external to, but not independent of, healthcare delivery.

**WHAT IS ALREADY KNOWN ON THIS TOPIC**

- Vaccination uptake often follows a socioeconomic gradient, with those who are most socioeconomically disadvantaged experiencing lower uptake.

**WHAT THIS STUDY ADDS**

- Provides insight into childhood vaccination programme delivery, from policy to practice, in an area of high socioeconomic disadvantage, yet with comparatively higher vaccine coverage.

**HOW THIS STUDY MIGHT AFFECT RESEARCH, PRACTICE OR POLICY**

- Encourages healthcare policy and practice professionals to target interventions toward promoting workforce stability, enabling trusting relationships between parents and providers to be more easily built. The impact of which would extend beyond childhood vaccination programmes.

## Introduction

 Inadequate coverage of childhood vaccinations is leaving children at risk of vaccine-preventable diseases (VPDs) and associated long-term complications.^1^ Globally, despite progress, many inequalities in vaccine supply, access and uptake remain.^1^ Differences in vaccination uptake exist between individuals and population groups, and can be associated with wider social inequalities.^2^ Vaccination inequalities affect a range of underserved populations, such as migrant and ethnically minoritised groups^3–6^; however, this study focuses on the socioeconomically disadvantaged, where unique trends in uptake are observed. While there is strong evidence for socioeconomic inequalities in vaccination uptake^7,8^—defined as lower coverage for disadvantaged groups—there can sometimes be ‘inverse’ associations: lower coverage for more advantaged groups and/or higher coverage for more disadvantaged groups.^9^ These ‘inverse’ associations are reportedly more evident in high-income countries.^10^

To improve childhood vaccination coverage and protect children from VPDs, it is pertinent to explore instances where coverage is (comparatively) higher despite the greater prevalence of socioeconomic disadvantage. In doing so, areas facing similar challenges of declining vaccination coverage, both nationally and internationally, can apply this learning to prevent further decreases. The North East of England consistently outperforms the other eight regions across five of the seven routine childhood vaccines, despite its higher prevalence of socioeconomic disadvantage.^10^ For example, the North East achieved 95.8% coverage for the 6-in-1 vaccine (diphtheria, tetanus, polio, pertussis, *Haemophilus influenzae* type B, and hepatitis B) among children aged 24 months in 2024–2025, compared with 94.4% in the South East and 87.1% in London. The North East has the highest prevalence of adults with no educational qualifications and the lowest healthy life expectancy of any region.^11^ Correspondingly, Middlesbrough, a local authority in the North East, has the highest rates of deaths related to suicide, alcohol and drug overdoses in the country,^12^ and the greatest proportion of socioeconomically disadvantaged neighbourhoods.^13^ The North East is the least populated region of England,^14^ and the least ethnically diverse, with 90.6% identifying as white British in 2021.^15^

There is a scarcity of published literature on vaccination equity in the North East, as most regionally focused studies examine London.^16–19^ However, a single qualitative interview study by Price *et al.*^20^ sought parents’ opinions on the barriers and facilitators to childhood influenza vaccination uptake in the North East; authors reported that the leading barriers were the lack of flexible appointment options and the low priority that parents with demanding schedules placed on vaccination. This suggests that the social determinants of health and personal circumstances play a significant role in delaying or preventing vaccination.^21^ Probing further into this from a service-delivery perspective would provide valuable insight into childhood vaccination uptake in a unique context, adding nuance to discussions around healthcare uptake and socioeconomic position by examining how vaccination providers in the North East understand local patterns in uptake and their role within them. To our knowledge, no other published study has explored this. Subsequently, this study aimed to explore programme providers’ perceptions of how the organisation, delivery, commissioning, support and monitoring of childhood vaccination may contribute to comparatively high uptake in the North East of England.

## Methods

The study adheres to Tong *et al.*’s^22^ COnsolidated criteria for REporting Qualitative research (COREQ). A completed copy of this checklist can be viewed in online supplemental material S1.

The research aims were addressed using qualitative semi-structured interviews: ‘planned but flexible’.^23 (p. 218)^ Flexibility was necessary so the interview guide could be adapted to resonate appropriately with the different professional roles of the interviewees.

### Sampling and recruitment

Purposive maximum variation and snowball sampling approaches were used. Eligible participants were health and care professionals who delivered, commissioned, monitored or supported the childhood vaccination programme in the North East of England. No eligibility restrictions were placed on, for example, years of career experience, length of time working in-post or any other criteria. The sampling frame reflected a multilevel systems approach and included the following roles, teams and organisations: (1) Regional childhood vaccination programme commissioners and childhood vaccination service delivery partners; (2) Governmental Local Authority Public Health Teams in the North East region. Participants from the regional and local authority levels were purposively selected from those who occupied senior oversight roles or had leadership over a relevant childhood-vaccination-related work package (eg, interventions to increase uptake); (3) In-practice delivery level, such as nurses, general practitioners (GPs), practice/business/operations managers working in areas of high socioeconomic disadvantage. These individuals were sampled from the North East and North Cumbria (NENC) Deep End (DE) Network. The NENC DE Network was created to support general practices in the region that serve patient populations experiencing high levels of socioeconomic disadvantage.^24^ It is recognised that GPs working in these areas face unique challenges compared with those serving less socioeconomically disadvantaged populations, such as lower staffing levels, caring for more patients per GP, and a greater prevalence of patients with complex needs.^25–30^

Relevant professionals, some of whom the research team had professional connections with, were contacted directly using email addresses. Additionally, publicly available general practice email addresses and the DE NENC member email mailing list were used to advertise the study, with a request to circulate to relevant employees. The study aimed to recruit 20 interviewees in total: 10 at the regional and local authority level and 10 at the provider level. However, recruitment was guided by the aim of capturing perspectives from key stakeholder groups and professional roles across the childhood vaccination system, and would cease before 20 interviewees if this was achieved.

### Data collection procedure

Video-calling software was used to conduct the interviews, and recordings were made using the built-in facilities. Online interviews were considered appropriate in view of: (1) the increased likelihood of familiarity and availability of video calling technology in the workplace and (2) to accommodate the restrictive and demanding working hours of health and care professionals. Research demonstrates that a successful rapport can be established with online methods.^31^

The interviews were conducted by AS, who was affiliated with the NENC DE Network Research Team at the time of data collection. This professional proximity may have facilitated rapport; however, it may also have made participants more inclined to present the strengths of local vaccination delivery and downplay criticism. AS’s familiarity with the local context may have also influenced the issues pursued in the interviews, as there was already a high level of NENC DE knowledge. The potential influence of this professional proximity on participant responses and data interpretation was considered during analysis and discussed with the wider project team, who were not affiliated with the NENC DE.

### Patient and public involvement

Patients and/or members of the public had no involvement in the design, conduct, reporting or dissemination of this study.

### Ethics

Prospective participants were provided with a participant information sheet to support informed decision-making, and a signed consent form was sought before the interview. The study adhered to the Data Protection Act 2018 and Newcastle University’s data storage and handling protocols. Interview recordings were deleted after the analysis was completed.

#### Interview schedule

The interview schedule was designed to last a maximum of 1 hour and began with introductory and background questions to establish rapport (see online supplemental material S2). Interview schedule development was informed by the literature on childhood vaccination uptake^9,32–34^ and other studies involving the NENC DE.^25,30,35,36^ For local authority and regional-level participants, the topics were first discussed in general, followed by a focus on areas of high socioeconomic disadvantage. Where appropriate, prompts and follow-up questions were used to probe further into responses.

### Data analysis

The interviews were transcribed verbatim. Names and other identifying information were removed, and the transcripts were labelled according to job role. NVivo V.14^37^ was used to facilitate the analysis, which took a framework approach developed for large-scale policy research^38^ (see online supplemental material S3). This method is appropriate when the researcher requires individual accounts to be easily identifiable, as the data are organised in a matrix table, with each row representing a participant and each column a specific code.^39^ These codes are then grouped to form themes, much like a standard thematic analysis. For our analysis, the codes were generated inductively. Gale *et al.’s*^39^ seven-step process for conducting framework analysis in multi-disciplinary health research was used as a guide.

The themes were discussed and refined among the four authors. To ensure the coding and theme formation processes were logical and appropriate, an independent researcher (TP) was sought as a secondary rater. TP has experience applying the framework approach to vaccination uptake studies.^20^ They independently coded one anonymised transcript, which was then compared with ours. There was a high degree of inter-rater consistency, with TP and ourselves identifying similar codes and agreeing on the subsequent theme development.

## Results

15 interviews were conducted between June 2023 and December 2023. There were no participant dropouts; however, the study advertisement received a low response rate, and one prospective participant declined due to work pressures. Two interviews had to be rescheduled due to technological issues. The participant details are presented in table 1; the ‘dual roles’ category refers to interviewees who were involved in the in-practice delivery of the childhood programme and also held a leadership role in the wider vaccination system. Accounts from participants occupying dual roles were analysed in the same way as those from participants in a singular role. 14 interviews were completed in 40–55 min, with one lasting over 1 hour.

**Table 1:** Characteristics of interview participants (N=15)

Participant details	Geographical location	Occupation details
In-practice delivery employees (n=5) (deliver the childhood vaccination programme)		
Nurse 1	Newcastle	Nurse practitioner
Nurse 2	Newcastle	
GP 1	Sunderland	GP and practice partner
Practice Manager 1	Middlesborough	Practice manager
Practice Manager 2	South Shields	
Dual roles (n=3) (deliver and support the childhood vaccination programme)		
GP 2	Gateshead	GP, practice partner and role in the leadership of external healthcare organisation.
GP 3	Sunderland	
GP 4	Durham	
Professionals involved in the wider childhood vaccination system (n=7) (commission, support and monitor the childhood vaccination programme)		
Commissioner 1	North East and Yorkshire	NHS England North East and Yorkshire
Commissioner 2		
Commissioner 3		
Commissioner 4		
Healthcare Delivery Partner 1	North East and Yorkshire	A private organisation that supports healthcare delivery.
Public Health Employee 1	Middlesborough	Local authority public health team.
Public Health Employee 2	Hartlepool	

GP, general practitioner; NHS, National Health Service.

After the first 13 interviews, all major actors in the childhood vaccination process (nurses, GPs, practice managers, commissioners, public health employees and healthcare delivery partners) were accounted for. Two more interviews were sought: one from a local authority public health employee to provide opinion diversity for this professional role, and one from a specific commissioner mentioned throughout the interviews as vital to the childhood vaccination system. Considering the multilevel approach to sampling, the aim was to capture perspectives from across the system rather than to comprehensively represent individual professional roles.

Three key themes were developed from the analysis: (1) the North East paradox - understanding childhood vaccination uptake rates in the region, (2) Challenges of delivering the childhood vaccination programme in areas with a high prevalence of socioeconomic disadvantage and (3) Parental vaccination knowledge and balancing informed choice against public health benefits.

### Theme 1: the North East paradox – understanding childhood vaccination uptake rates in the region

Uptake in the North East outperformed any other region in England for most childhood vaccines; reportedly, this is a historical trend.

We’ve actually been like this in the North East for many years…been either at or near the top of the coverage rates in the country*.* (Commissioner 3)

Interviewees offered many hypotheses for this trend. However, Commissioner 2 and Healthcare Delivery Partner 1 were keen to emphasise that these were based on their personal opinions and experiences, not quantitative, statistical evidence. The concept of ‘stability’ underpinned many of these hypotheses. It was first suggested that the North East has a less mobile population and ‘more stable primary care, and maybe even [a] better resourced primary care system’ (Commissioner 3) than in other regions, such as London.

For communities, stability promotes ‘next-door neighbour knowledge’ (Commissioner 1) that can encourage parents to vaccinate their children. Commissioner 1 animated their example by mimicking a North East parent: ‘Eee, my sister had measles, and it was awful. You want to get them [children] jabbed!’ Subsequently, this perpetuates the idea that vaccinating your child is a ‘cultural thing’ [Delivery Partner A], a social norm of the region; ‘there is just a history of you get your jabs’ [Delivery Partner A].

For local healthcare services, stability means that trusting relationships between patients and providers are more easily built. Participants discussed the national decline in patient–provider relations and the decreased prevalence of a named, family GP. Commissioners reported feeling that this decline in relations was perhaps to a lesser extent in the North East than in other regions. Additionally, from a data-monitoring perspective, it is easier in less mobile populations to ensure that child health records are up to date. Transferring child health records to another Child Health Information Service team is not always seamless due to different operating systems.

…it’s also quite a stable population; if you look at somewhere like London, they’ve got a huge amount of movement of population, and that can’t be easy in terms of those very fundamental measures of having a consistent, trusted relationship between primary care and population, and just keeping records straight, making sure that those records follow the people… (Commissioner 3])

Another explanation for the higher levels of coverage in the North East was the reported lack of population groups that face more ‘barriers’ (Healthcare Delivery Partner 1) to accessing healthcare services than other populations. One of these populations is ethnically minoritised communities, who are reportedly underrepresented in the North East.

…we are the least ethnically diverse part of England. We know the uptake is lower in minority and ethnic groups… (Healthcare Delivery Partner 1)

Nevertheless, Healthcare Delivery Partner 1 and the commissioners who commented on this topic suggested that the current healthcare system in the North East may underserve these communities because their adverse experiences are not adequately addressed.

It’s whether or not we have rested on our laurels because we’ve had such high uptake that, actually, we haven’t adapted our delivery to meet the changing demographic. That would be a failing on healthcare services, to not engage communities in culturally sensitive and competent ways*.* (Healthcare Delivery Partner 1)

Commissioners described concerns with the narrative that the North East demonstrates comparatively higher levels of uptake. First, they highlight that the area has seen the same recent post-COVID-19 ‘gradual reduction in uptake that they've seen across the rest of the country’ (Commissioner 1). Second, on closer inspection, the narrative does not necessarily reconcile at a more granular level.

I think that what happens with the ‘we get good rates’ is that you actually miss out pockets of the population; you don’t look hard enough*.* (Commissioner 1)

Newcastle City and Middlesbrough were referenced as two of these low-uptake areas. One possible explanation for this was that Newcastle City and Middlesbrough have high levels of mobile communities and ethnic diversity.

There’s a lot of mobile communities and ethnic groups within Newcastle, similarly within Middlesbrough, so those are the areas that we struggle with uptake particularly. (Commissioner 2)

This explanation aligns with previous discussions on stability and population demographics.

Some interviewees attributed Middlesbrough’s lower uptake to its high prevalence of socioeconomic disadvantage. Yet, it was simultaneously recognised that less disadvantaged areas can exhibit lower coverage, especially of the measles, mumps and rubella (MMR) vaccine.

I think Middlesbrough is one of our most deprived…so that may have something to do with it. Having said that, we don’t really have a large body of evidence that directly links deprivation with poor uptake in childhood imm[unisation]s. In particular, we looked at MMR, and actually, it didn’t prove that at all. There are some areas that are really quite affluent who have low uptake, and vice versa*.* (Commissioner 2)

Equally, some interviewees who worked in general practices in socioeconomically disadvantaged areas insisted their uptake levels were not low; an insinuation supported by those from the wider vaccination system. However, ‘what they were having to do to secure those, was over and above’ (Commissioner 1). It was felt that general practices located in socioeconomically disadvantaged areas had to invest more staff time and effort into meeting vaccination uptake targets than practices in less disadvantaged areas.

To get my immunisation targets, as I described earlier, it takes a lot more effort than in the kind of practice where everybody just turns up and gets their jabs*.* (GP 3)

### Theme 2: challenges of delivering the childhood vaccination programme in areas with a high prevalence of socioeconomic disadvantage

Most children are vaccinated within the recommended timeframe, with minimal intervention from their general practice. For some, however, more input is required. This holds especially true for children from underserved communities, of which there is reportedly a higher prevalence registered at general practices in areas of high socioeconomic disadvantage. One group that required more general practice intervention to attend for childhood vaccinations were those from ‘chaotic families’ or ‘patients with chaotic lives’, to which 12 of the 15 participants referred.

…immunisations are always more difficult in populations who find it hard to bring organisation into quite chaotic lives…because crises happen regularly in their lives and that takes precedence over a planned attendance*.* (GP 3)

Interviewees conceptualised ‘chaotic families’ differently, but the term was often used in a safeguarding context.

Usually it relates to alcohol, drugs, police involvement, crime…or domestic violence in the home…these families frequently have safeguarding or child protection issues, are children-in-need, or are looked-after children*.* (Nurse 1)

Some participants questioned the term ‘chaotic families’ to describe this group, because it is ‘just blaming those families, when maybe we could be more person-centred in our approach to healthcare delivery’ (Public Health Employee 2). As a consequence of the difficulties these families are experiencing, they may face an incompatibility with planned healthcare and thus struggle to schedule and attend their child’s vaccination appointments.

…I don’t think it’ll be her priority to say, ‘In 8 weeks, I’m going to come in. Have I got that appointment? Is my red book ready?’ And all the things that probably, if you’re a middle-class person who your health and your baby’s health is really on your mind all of the time, everything’s meticulous… (GP 2)

However, GP 4 takes a different approach to this subject, rejecting the undertones of blame and stating, ‘if you’ve not got the basis of the warm house or roof over your head, you’ve not got food on the table, then vaccination is not going to be a priority’ (GP 4). In the above quotes, GP 2 and GP 4 use the term ‘priority’, which does not imply negative opinions about vaccination, but suggests that other personal issues may be more imminent and concerning. This narrative was supported by other interviewees.

…it’s not even probably about choosing to have them and not choosing to have them, just never getting around to it because there’s so much else going on in their life that they're preoccupied by. (Public Health Employee 1)

To encourage vaccination appointment attendance, these families ‘need the more personal touch, and not just personal, but continuity’ (GP 2).

Commissioner 3 captures the essence of theme two in the following quote:

It’s the Pareto effect, isn’t it? It’s quite easy to get the first 80 or 90% just by doing the routine things, but you need to put more effort into that last 5 or 10%*.*

### Theme 3: parental vaccination knowledge and balancing informed choice against public health benefits

The third theme reflects on the wider social processes of vaccine and vaccination knowledge and their intersection with the childhood programme.

Lack of VPD awareness is reportedly an increasing national public health issue. Due to the success of the vaccination programme in reducing disease prevalence, a large proportion of the UK population has not witnessed the potential impacts and long-term implications of contracting VPDs. Subsequently, ‘a little bit of complacency has probably snuck in’ (Public Health Employee 1). Two participants referred to the childhood programme being a ‘victim’ (Public Health Employee 2 and Commissioner 2) of its success. Similarly, it was believed that some parents may be unaware of why multiple doses of the same antigen are required, and thus, view the schedule as a ‘pick ‘n’ mix’ (Public Health Employee 1), preventing full immunity from developing. Interviewees who had worked in public health or healthcare provision for decades recalled the risks of not vaccinating.

…in my lifetime, I’ve seen children going blind, I’ve seen children being disabled from vaccine-preventable diseases, very few parents have. And sometimes I would say that, ‘I know this is really, really difficult, but this is what these diseases can do’. (Public Health Employee 2)

However, when parents decide not to vaccinate, it is important to ensure these decisions are informed, meaning parents understand the risks of not doing so. Healthcare Delivery Partner 1 stressed this fact: ‘What I don’t want people to have an ill-informed, or not informed, decision not to allow their child to be vaccinated*.’* Childhood vaccination is unique among other healthcare interventions because the implications extend beyond the decision-maker to the child and the wider population through herd immunity.

…people should be able to have a choice, but the other part of me thinks: the child doesn’t have a choice; it’s somebody else making that choice for them. I mean, if you went blind because your mum didn’t get your measles… (Practice Manager 1)

Interviewees from the wider vaccination system reported a unique tension between parents who decide not to vaccinate their children and the population’s health, sparking debates about individual liberty in a UK context.

Do we say, ‘Well, the most important thing is people get to make a choice!’, or do we say, ‘Well, actually, all these people making a choice have now put all these people at risk, because we don’t have herd immunity anymore because they didn’t feel it was right for their child.’ It’s looking at how you manage that because, ethically, you don't want to take people’s choice away, but on the other hand. (Commissioner 1)

As with any pharmacological intervention, there is always the risk of adverse effects and reactions. However, it was suggested that parents view vaccines differently from other pharmacological interventions because they are (primarily) administered through injection. One interviewee believed this was because immunisation via injection was perceived as more ‘potent’ and ‘irrevocable’ (Public Health Employee 2) by parents than, for instance, the childhood influenza vaccine administered via nasal spray.

Some parents don’t have the confidence to deal with their child receiving an injection; that’s very difficult. Whereas the nasal drop doesn’t seem as bad. That they’re [injections] hurting them, that it may do something, the links with Autism, and that we’re damaging this perfect baby that they’ve given birth to*.* (Public Health Employee 2)

However, to overcome these concerns of parents, Commissioner 3 emphasised that parent-provider relations were crucial, due to the ‘power of the trusted voice’.

## Discussion

This study aimed to explore programme providers’ perceptions of how the organisation, delivery, commissioning, support and monitoring of childhood vaccination may contribute to comparatively high uptake in the North East of England. Overall, interviewees felt that the higher childhood vaccination coverage in the North East of England was explained not by the approach to provision but by the region’s sociocultural, demographic and healthcare system characteristics. The North East region was hypothesised to be more stable and less mobile in terms of both its population and healthcare workforce; conditions under which trusting parent–vaccinator relationships are more easily built and maintained. Despite this, general practice staff working in areas of high socioeconomic disadvantage described challenges, including families with complex personal circumstances and parents not viewing vaccination as a priority. In these areas, it was felt more resources were required to achieve and maintain uptake levels. Interviewees offered broader reflections on the growing complacency regarding the potential health impact of VPDs and the importance of trusted voices in addressing parents’ vaccination concerns.

This study further demonstrates the complex association between socioeconomic factors and childhood vaccination uptake; namely, trends may not reflect a clear gradient as observed with other healthcare interventions, such as cancer screening.^40,41^ The North East illustrates how a region can achieve high vaccination coverage despite a high prevalence of socioeconomic disadvantage; however, this may not reflect the situation at a more granular level. While the North East is often characterised as having relatively low cultural diversity, this regional picture can obscure important local variation. Providers working in highly deprived areas described serving more culturally diverse communities, reflecting the concentration of some ethnic minority groups within socioeconomically disadvantaged localities. This is important because these communities may face multiple, overlapping barriers to vaccination uptake, while also living in areas where healthcare services are already under substantial pressure. These patterns reflect wider structural inequalities.^42,43^

Similarly, structural-level barriers for families with complex personal circumstances of social and economic disadvantage were often attributed by providers in these areas to individual-level social determinants,^44^ such as vaccination not being a ‘priority’ for some parents. Indeed, this sentiment aligns with Price *et al.*’s^20^ study on parental opinions of childhood influenza vaccination uptake in the North East of England, as discussed in the introduction. Other research has emphasised the role of ‘day-to-day socio-material circumstances’^(45, p. 239)^ on vaccination uptake, encouraging healthcare policy and practice professionals to focus on reducing access barriers.^9,45,46^ However, the term ‘chaotic families’, used by interviewees to describe those with complex personal circumstances, places blame on individuals, reflecting a similar narrative to that previously discussed in relation to the concentration of ethnic minority communities in socioeconomically disadvantaged areas. Namely, that healthcare services in these areas are more likely to be understaffed and overwhelmed.

References to ‘stability’ and ‘trust’ occurred throughout the analysis. Trust and stability can foster safe environments for parents and providers to discuss childhood vaccination. Indeed, having a trusted source of vaccination information^9,47–49^ and a named, consistent healthcare provider^50,51^ are both promoters of vaccination uptake. Our results suggest this relationship is not only important for overcoming vaccine hesitancy but also for encouraging parents to view vaccination as a priority. This can be understood through theories of social capital that posit how local networks, relationships and shared norms may support engagement with healthcare.^52^ However, the COVID-19 pandemic resurfaced concerns regarding vaccination,^48,53^ and trust in scientific expertise and public health authorities is declining.^51^ These processes have been referred to as a ‘double-edged sword’^54^ because vaccines were pivotal in curtailing the pandemic’s impact but were met with considerable scepticism and apprehension. Indeed, social capital is not inherently positive, as local networks can reproduce misinformation and negative views of vaccination, contributing to a norm of vaccine hesitancy and scepticism. Looking to the future, experts in the field insist that, globally, trust in healthcare providers must improve for vaccination uptake to follow suit.^55^ This is vital, as research suggests that, without intervention, global childhood vaccination uptake will no longer be on a continuous upward trajectory as in previous years.^56^ However, building trust is challenging for parents and healthcare providers in socioeconomically disadvantaged areas when the structural challenges they both experience are not recognised and effectively navigated. In summary, vaccination uptake appears to be substantially influenced by social processes external to, but not independent of, healthcare delivery.

### Strengths and limitations

This study provides unique insight into the delivery of the childhood vaccination programme in the North East of England, an area of high socioeconomic disadvantage but with comparatively higher vaccination uptake. Which, to our knowledge, no other published study has yet explored. We highlight the importance of healthcare workforce stability as a prerequisite for repairing parent–vaccinator trust; other studies discuss these concepts in isolation, but not their interaction in the context of a high-uptake, high-socioeconomic-disadvantage area.

However, there are limitations. Vaccination service providers were recruited only from NENC DE general practices which serve communities experiencing high socioeconomic disadvantage. As such, there is a risk we have only included the accounts of those with similar opinions. Although this was the focus of our research, for comparison, we could have sampled from outside the Network from other general practices serving communities experiencing varying levels of socioeconomic disadvantage.

An external researcher (TP) independently coded one anonymised transcript, which was then compared with our findings. Although there was a high degree of inter-rater consistency, with both TP and AS identifying similar codes and agreeing on the development of themes, it could be argued that double-coding a single transcript does not provide sufficient rigour. However, a framework approach provides a transparent and comprehensive overview of the analysis process, and the development of the themes was discussed among the authorship team.

### Recommendations for policy and practice

Two main recommendations for policy and practice arose from this study. Interventions to improve vaccination uptake should consider healthcare stability as a prerequisite to repairing and maintaining parent–provider trust. Trusting your child’s vaccination (and healthcare) provider is crucial in highlighting the importance of vaccination (and preventative healthcare interventions), ensuring timely uptake, and overcoming concerns. Without this, coverage will continue to decline, and outbreaks of VPDs will become increasingly difficult to prevent. While the transferability of these findings should be considered in relation to the specific contextual conditions of the North East, they remain useful in highlighting how workforce stability and established service relationships may support parent–provider trust in socioeconomically disadvantaged communities.

Second, the importance of having a detailed understanding of socioeconomic inequalities in vaccination uptake cannot be overstated. It is recommended, globally, that all levels of the system (national, regional, neighbourhood, and primary care) be intimately aware of how socioeconomic inequalities in vaccination uptake manifest in their respective populations. Interventions to improve uptake must be appropriately targeted at groups with suboptimal uptake.

## Conclusion

Although childhood vaccination coverage is comparatively high across North East England, programme providers described substantial variation between and within localities, suggesting that regional averages may mask communities experiencing lower uptake and barriers to vaccination. Interviewees felt that the higher childhood vaccination coverage in the region was explained not by the approach to provision but by the area’s sociocultural, demographic and healthcare system characteristics. Providers’ accounts indicated that the organisation and delivery of services may shape uptake at the local level, particularly through accessibility, continuity of care, knowledge of local communities and trusting relationships between families and vaccination staff. Greater workforce stability may help support continuity and trust. Monitoring uptake at a sufficiently granular level, and tailoring service delivery to the circumstances of socioeconomically disadvantaged communities, may therefore be necessary to identify and address inequalities obscured by comparatively favourable regional coverage.

## Supplementary material

10.1136/bmjph-2026-005188 online supplemental file 1

## Data Availability

No data are available.
