# Supplementary material for: Programme providers’ perceptions of local variation in childhood vaccination uptake in a socioeconomically disadvantaged UK region: a qualitative study
Source: BMJ Public Health. 2026 Oct 22;4(4):e005188. doi: 10.1136/bmjph-2026-005188 (PMC13691774; doi:10.1136/bmjph-2026-005188)
Supplement: Supplementary file 1 [file bmjph-4-4-s001.pdf]

Supplementary File

Increasing the uptake of childhood vaccines: Insights from qualitative interviews with programme providers in a high-uptake socioeconomically disadvantaged region of the UK,

Sacre *et al*.

S1: CoreQ Checklist

No.	Item	Guide questions/description	Relevant page/Notes
**Domain 1: Research team and reflexivity**			
**Personal Characteristics**			
1.	Interviewer/facilitator	Which author/s conducted the interview or focus group?	Dr Amber Sacre, PhD
2.	Credentials	What were the researcher's credentials? E.g. PhD, MD	PhD in Population Health Sciences
3.	Occupation	What was their occupation at the time of the study?	Post-doctoral research associate
4.	Gender	Was the researcher male or female?	Female
5.	Experience and training	What experience or training did the researcher have?	Essex Summer School of Qualitative Data Analysis
**Relationship with participants**			
6.	Relationship established	Was a relationship established prior to study commencement?	p. 7/8
7.	Participant knowledge of the interviewer	What did the participants know about the researcher? E.g., personal goals, reasons for doing the research	p. 7/8
8.	Interviewer characteristics	What characteristics were reported about the interviewer/facilitator? E.g., bias, assumptions, reasons and interests in the research topic	p. 6
**Domain 2: Study design**			
**Theoretical framework**			
9.	Methodological orientation and theory	What methodological orientation was stated to underpin the study? E.g., grounded theory, discourse analysis, ethnography, phenomenology, content analysis	Case study approach – pre-implementation evaluation
**Participant selection**			
10.	Sampling	How were participants selected? E.g., purposive, convenience, consecutive, snowball	p. 7/8
11.	Method of approach	How were participants approached? E.g., face-to-face, telephone, mail, email	p. 7/8

12.	Sample size	How many participants were in the study?	p. 9
13.	Non-participation	How many people refused to participate or dropped out? Reasons?	p. 9
**Setting**			
14.	Setting of data collection	Where was the data collected? E.g., home, clinic, workplace	p. 6/7
15.	Presence of non-participants	Was anyone else present besides the participants and researchers?	No
16.	Description of sample	What are the important characteristics of the sample? E.g., demographic data, date	p. 7
**Data collection**			
17.	Interview guide	Were questions, prompts, guides provided by the authors? Was it pilot tested?	Supplementary material and p. 6
18.	Repeat interviews	Were repeat interviews carried out? If yes, how many?	N/A
19.	Audio/visual recording	Did the research use audio or visual recording to collect the data?	p. 6/7
20.	Field notes	Were field notes made during and/or after the interview or focus group?	No
21.	Duration	What was the duration of the interviews or focus group?	p. 6
22.	Data saturation	Was data saturation discussed?	p. 8-10
23.	Transcripts returned	Were transcripts returned to participants for comment and/or correction?	No
**Domain 3: analysis and findings**			
**Data analysis**			
24.	Number of data coders	How many data coders coded the data?	p. 6
25.	Description of the coding tree	Did authors provide a description of the coding tree?	Supplementary Material S3
26.	Derivation of themes	Were themes identified in advance or derived from the data?	Generated in advance, but iteratively developed
27.	Software	What software, if applicable, was used to manage the data?	p. 8
28.	Participant checking	Did participants provide feedback on the findings?	No
**Reporting**			
29.	Quotations presented	Were participant quotations presented to illustrate the themes/findings? Was each quotation identified? E.g., participant number	p. 11-17
30.	Data and findings consistent	Was there consistency between the data presented to illustrate themes/findings?	Yes
31.	Clarity of major themes	Were major themes clearly presented in the findings?	Yes

32.	Clarity of minor themes	Is there a description of diverse cases or discussion of minor themes?	Yes

S2: Interview Guide

Overview:

- Describe the state of childhood vaccination uptake.
- Challenges to delivering/commissioning/supporting/monitoring the childhood vaccination programme.
- Successes delivering/commissioning/supporting/monitoring the childhood vaccination programme.

Specific:

- Describe local patient needs.
- Describe difficult-to-navigate policies and/or procedures.
- Describe the data monitoring process and involvement.

S3: Framework analysis exemplar.

	**Initial* Theme 1**	**Initial* Theme 2**	**…**
**Transcript 1**	*Initial* Codes: A.1, A.2, A.3*<br>• Summarise the contents of transcript 1 relevant to initial Theme 1.<br>• *"relevant quote/s"*	*Initial* Codes B.1, B.2, B.3*<br>• Summarise the contents of transcript 1 relevant to initial Theme 2.<br>• *"relevant quote/s"*	
**Transcript 2**	*Initial* Codes: A.1, A.2, A.3*<br>• Summarise the contents of transcript 2 relevant to initial Theme 1.<br>• *"relevant quote/s"*	The cell remains blank if the transcript does not refer to the theme.	
**…**			

**Themes and codes undergo several iterations to best reflect the data.*
